# Supplementary material for: Spatiotemporal terahertz emission nanoscopy of spintronic photocurrent
Source: Light Sci Appl. 2026 Oct 8;15:386. doi: 10.1038/s41377-026-02468-2 (PMC13645728; doi:10.1038/s41377-026-02468-2)
Supplement: Supplementary file 1 [file 41377_2026_2468_MOESM1_ESM.pdf]

Supplementary Information for

Spatiotemporal Terahertz Emission Nanoscopy of Spintronic Photocurrents

F. Paries*†[1,2], R. Rouzegar*[3], J. Cai[4,5], M. Dai[4,5], F. Selz[1,2,6], J. Koelbel[6], G. Lezier[7], N. Tiercelin[7], D. Molter[2], D. M. Mittleman[6], G. von Freymann[1,2], X. Wu†[4,5,8], and T.S. Seifert†[3]

*equal contributions
†corresponding authors

[1] Department of Physics and Research Center OPTIMAS, RPTU Kaiserslautern-Landau, 67663 Kaiserslautern, Germany
[2] Fraunhofer Institute for Industrial Mathematics ITWM, 67663 Kaiserslautern, Germany
[3] Department of Physics, Freie Universität Berlin, 14195 Berlin, Germany
[4] Hangzhou International Innovation Institute, Beihang University, Hangzhou 311115, China
[5] School of Electronic and Information Engineering, Beihang University, Beijing, 100191, China
[6] School of Engineering, Brown University, Providence RI 02912 USA
[7] University of Lille, CNRS, Centrale Lille, Université Polytechnique Hauts-de-France, UMR 8520 - IEMN, 59000 Lille, France
[8] Zhangjiang Laboratory, 100 Haike Road, Shanghai 201204, China

Experimental setup

As depicted in Fig. S1, the output of an Erbium-doped femtosecond laser (100 MHz repetition rate) is split into two trains of pump and probe pulses, each amplified by an Erbium-doped fiber amplifier (EDFA) to an average power of 20 mW. To stay below the damage threshold of the single-mode fiber-tip[1], the pump arm is attenuated to 2 mW using a variable optical attenuator. The fiber-tip STE is integrated into a commercial THz SNOM system (*attocube*) using a custom holder mounted on the system's three-axis piezo stage. The nanoscopic THz SNOM tip emits a terahertz field into the optical far field that is modulated at the tip's 48 kHz tapping frequency. A parabolic mirror collimates the radiation before it is focused by a Silicon lens onto a photoconductive antenna, that becomes transiently conductive upon probe-pulse excitation. The THz-induced photocurrent is transimpedance amplified and the resulting signal lock-in detected. By imposing a varying time delay between the THz and the probe pulse, the terahertz waveform is sampled in the time domain. Notably, the THz signal bandwidth is not limited by the PCA detector but rather by the signal-to-noise ratio. THz SNOM setups inherently favor lower terahertz frequencies, since the tip and the tip-sample junction act as a low-pass filter[2,3].

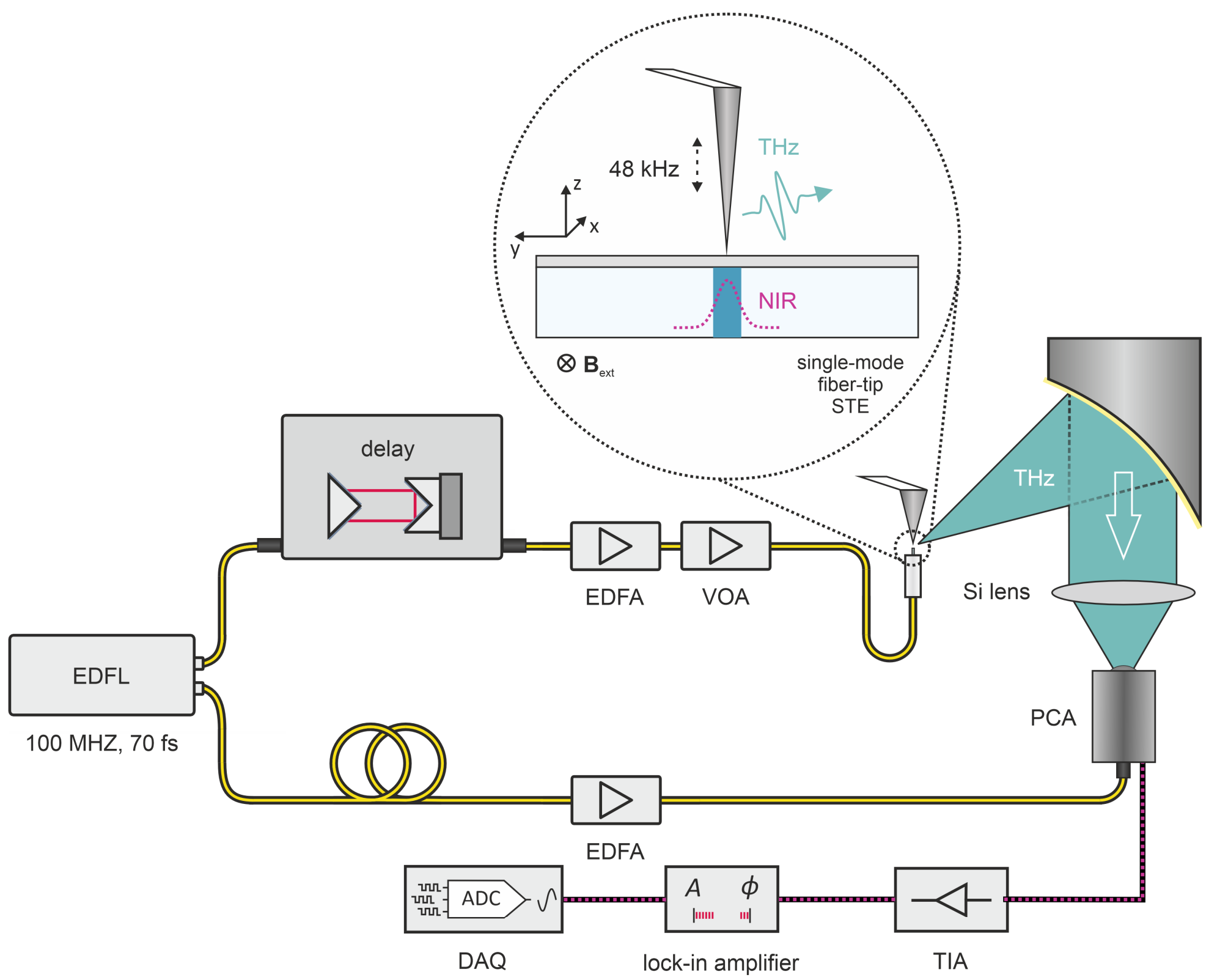

Fig. S1 | Illustration of the experimental setup. A single-mode fiber-based optical sampling setup is combined with a fiber-tip spintronic terahertz emitter and a THz SNOM system. EDFL – Erbium-doped fiber laser, EDFA – Erbium-doped fiber amplifier, VOA – variable optical attenuator, PCA – photoconductive antenna, TIA – transimpedance amplifier, DAQ – data acquisition.

Simulated charge-current densities and electric-field components

Upon absorption of the femtosecond pulse, a transient spin current is injected from the ferromagnetic layer into the adjacent heavy-metal layers and converted into a primary in-plane charge current perpendicular to the magnetization direction of the ferromagnetic layer. This primary in-plane charge-current density $\mathbf{j}_{\text{primary}}$ follows the spatial intensity profile of the gaussian-shaped pump-field. Due to the layer's metallic nature with a conductivity of approximately 10^6 S m^{-1}, the sheet conductance of the spintronic THz emitter is sufficiently high to allow for a significant induced currents despite the small

thickness of 6 nm according to Ohm's law. The sum of the primary charge-current density $\mathbf{j}_{\mathrm{primary}}$ and this induced charge-current density $\mathbf{j}_{\mathrm{ind}}$ is visualized in Fig. S2a.
Since the size of the primary Gaussian charge-current-density profile, and thus the scale over which the in-plane electric-field components E_{x} and E_{y} spatially vary, is comparable to or smaller than the THz wavelength inside the material, a significant out-of-plane electric field component emerges that can couple efficiently to a metallic SNOM tip (Fig. S2b).

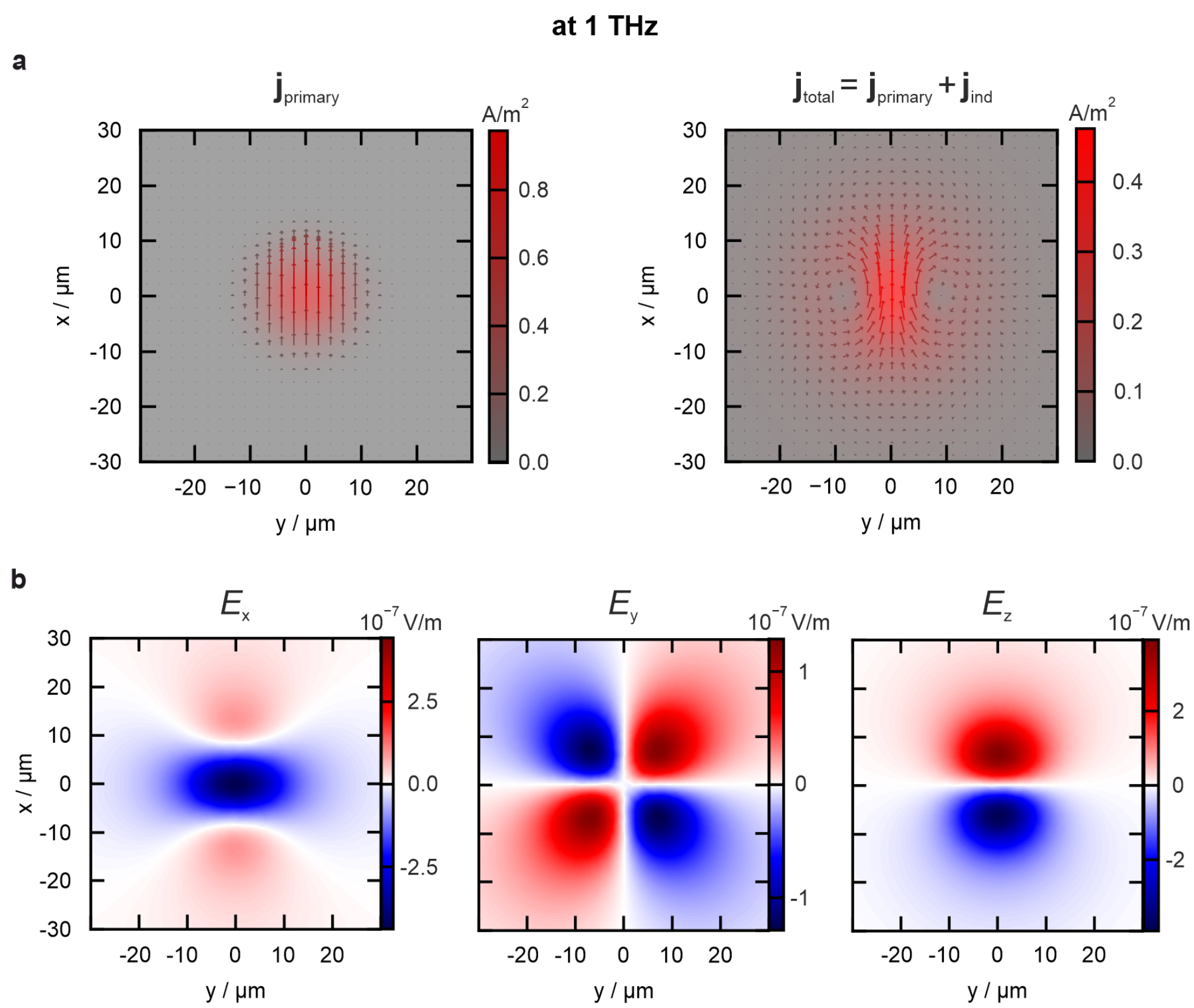

Fig. S2 | Simulated charge-current densities and electric-field components. a Vector-field representation of the simulated primary charge-current densities $\boldsymbol{j}_{primary}$ and simulated total charge-current densities $\boldsymbol{j}_{total} = \boldsymbol{j}_{primary} + \boldsymbol{j}_{ind}$, where $\boldsymbol{j}_{primary}$ maps the gaussian pump-mode field profile and $\boldsymbol{j}_{ind}$ is the induced charge current governed by the material properties. Turning on the metallic thin-film properties ($\boldsymbol{j}_{primary} + \boldsymbol{j}_{ind}$), two vortices emerge. The color mapping visualizes the absolute value of the vector field. **b** Simulated electric-field distributions related to the current densities in a.

Electric-field enhancement

We calculate the effect of field enhancement of the tip by simulating an STE with a tip close to the metallic film. A metallic tip with an apex of 100 nm and conductivity of $\sigma = 1.2 \times 10^7$ is considered. We observe that E_z is enhanced by about an order of magnitude within the vicinity of the metallic nanoscopic tip as showcased in the simulated electric field in Fig. S3. This causes a charge-current density to be induced within the tip that is similar in magnitude as the primary in-plane current inside the STE.

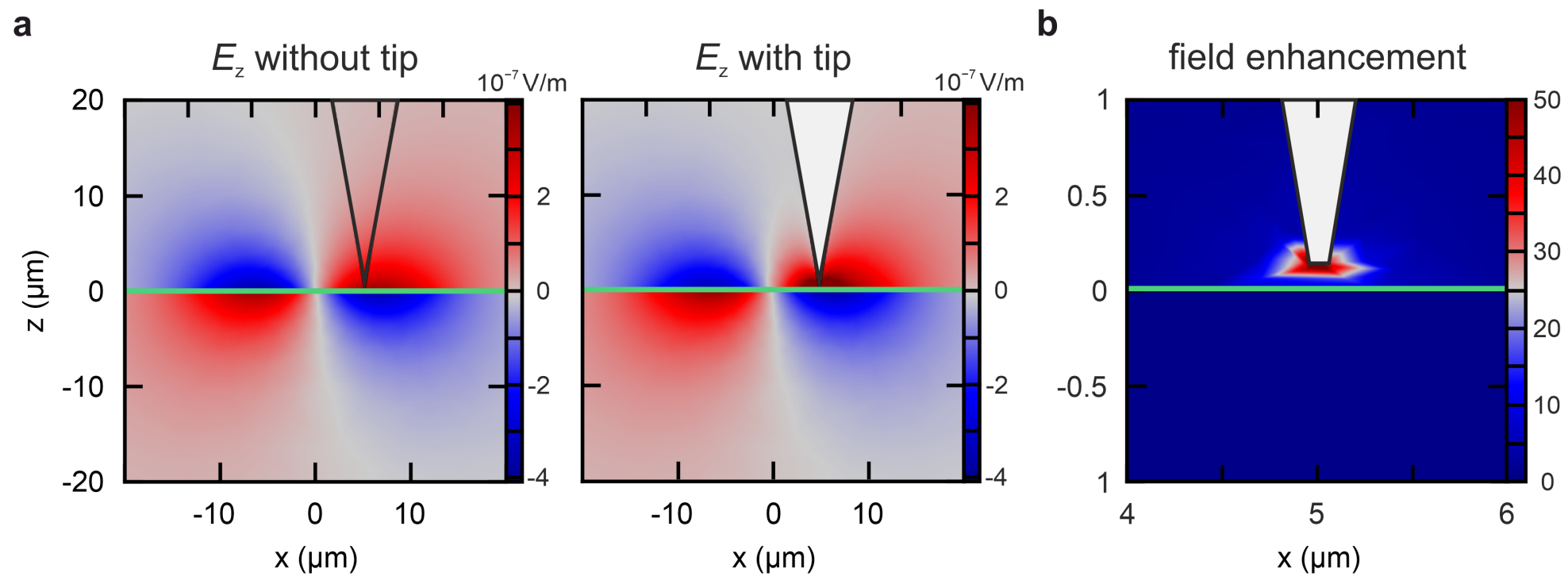

Fig. S3 | a Simulated E_z without and with the presence of a sharp metallic tip. The STE is illustrated in green. **b** Visualization of the tip-induced light-field enhancement. The out-of-plane electric field is enhanced by more than one order of magnitude. The edgy appearance is caused by the limited resolution of the simulation mesh.

Conductance dependence

In this section, we investigate the effect of film thickness on the E_z component responsible for the measured THz signal. In practice, increasing the film thickness reduces the absorbed pump energy density. However, since our goal is to isolate the dependence of E_z on film thickness, we normalize all simulations to the same absorbed energy density. Equivalently, the excitation strength (and thus j_0) is kept identical for all thicknesses.

We fix the conductivity at $\sigma = 10^3$ S m^{-1} and vary the film thickness between $d =$ 5, 10, and 20 nm. The simulations show that E_z increases with increasing thickness. When this result is combined with Fig. 5b of the main manuscript, which shows that E_z increases with σ, we conclude that E_z scales with the film conductance ($G = \sigma d$) rather than with the bulk conductivity alone.

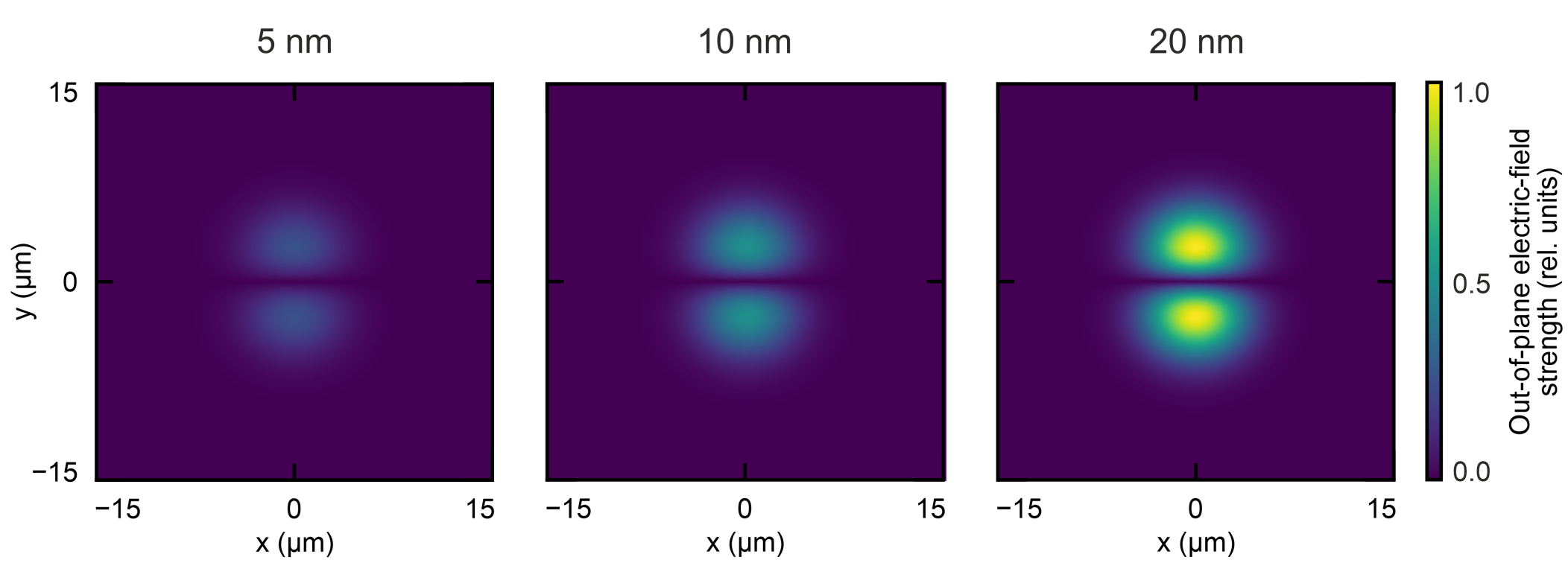

Fig. S4 | Simulated maps of the out-of-plane electric field E_z (ω, x, z). Dependence of E_z on the film thickness while keeping conductivity $\sigma = 10^3$ S m^{-1} and energy density (or equivalently same primary current) same for all thicknesses

Correction of periodic instrument drifts

Fig. S5 illustrates the delay-correction procedure described in *Materials and methods.* Repetitive temperature variations on the time scale of hours result in thermal expansions and contractions that cause the piezo-stage control loop to adjust the z-axis position during the measurement to keep the average tip-sample distance constant. The z-channel readout thus provides a calibration signal to correct for the resulting periodic delay shifts.

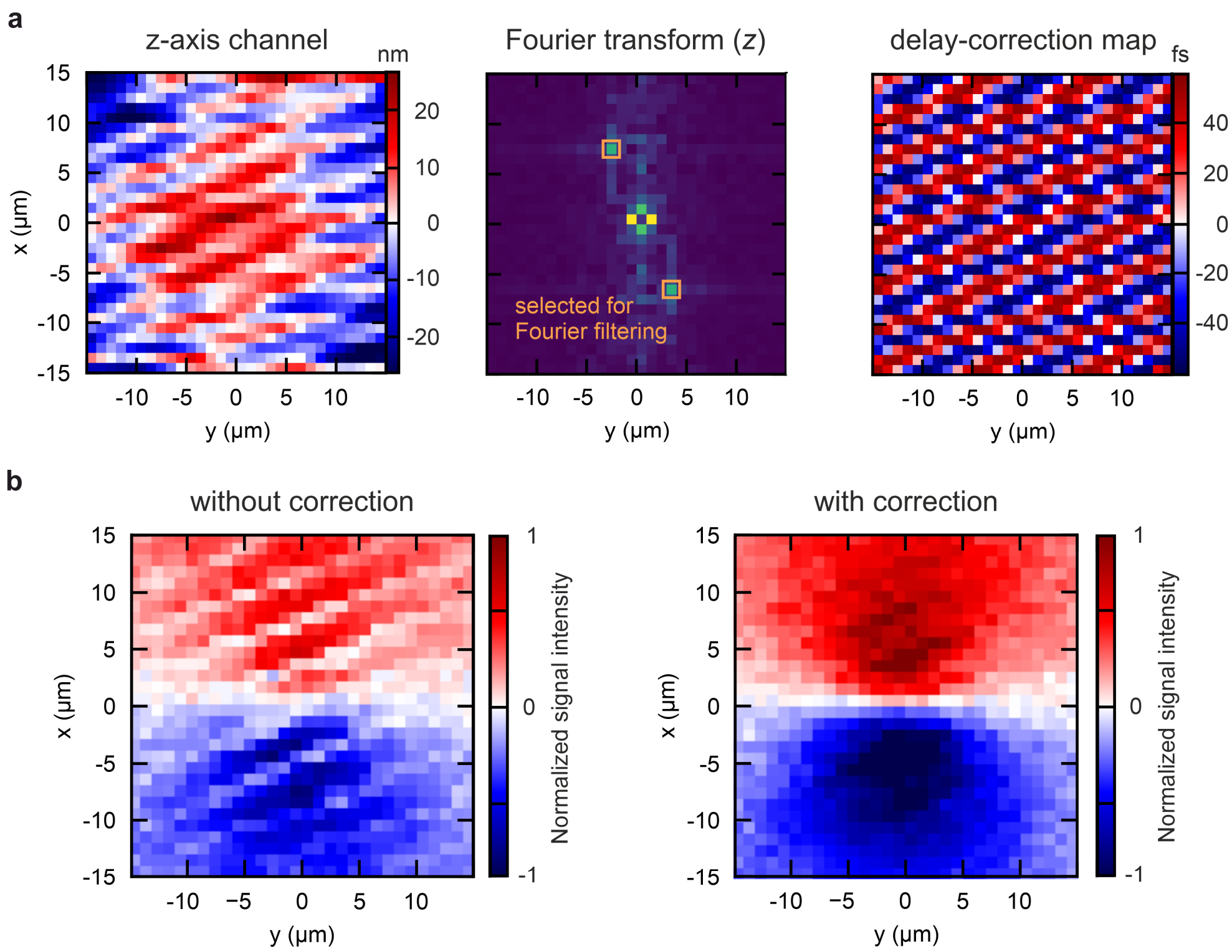

Fig. S5 | Correction of periodic instruments drifts. a A delay-correction map is created by applying spatial Fourier filtering to the z-channel data and setting the amplitude to 56 fs. **b** Acquired terahertz maps at a fixed delay before and after applying the delay-correction map.

References

1. Paries, F. et al. Optical damage thresholds of single-mode fiber-tip spintronic terahertz emitters. *Optics Express* **32**, 24826-24838 (2024).
2. Guo, X. et al. Terahertz nanoscopy: advances, challenges, and the road ahead. *Applied Physics Reviews* **11**, 021306 (2024).
3. Müller, M. et al. Phase-resolved detection of ultrabroadband THz pulses inside a scanning tunneling microscope junction. *ACS Photonics* **7**, 2046-2055 (2020).
